# Alcohol Use and Preventing Alcohol-Related Problems Among Young Adults in the Military

**Published:** 2004

**Authors:** Genevieve Ames, Carol Cunradi

**Affiliations:** Genevieve Ames, Ph.D., is a senior research scientist, and Carol Cunradi, M.P.H., Ph.D., is a research scientist, both at the Prevention Research Center, Pacific Institute for Research and Evaluation, Berkeley, California

**Keywords:** military, military personnel, young adult, heavy drinking, binge drinking, causes of AODU (alcohol and other drug use), AODU susceptibility, work-related AOD issue, cultural patterns of drinking, AOD availability, workplace-based prevention, prevention of problematic AODU, targeted prevention, prevention campaign, alcoholic beverage distribution laws, expectancy, personal responsibility, health promotion

## Abstract

Heavy alcohol use is a significant problem in the military. Personnel often use alcohol in an attempt to cope with stress, boredom, loneliness, and the lack of other recreational activities. The easy availability of alcohol, ritualized drinking opportunities, and inconsistent policies contribute to a work culture that facilitates heavy and binge drinking in this population. Prevention strategies such as alcohol use policies combined with campaigns focusing on alcohol deglamorization, personal responsibility, and health promotion currently are being implemented to help reduce heavy alcohol use, but further research is needed to evaluate the effects of these efforts. Understanding the characteristics of military culture that encourage or allow heavy and binge drinking practices also will help in designing effective prevention approaches.

Relative to other substance use, heavy drinking (i.e., consuming five or more drinks per typical drinking occasion at least once a week) appears to be a particularly persistent problem in the military. Although illicit drug use and cigarette smoking both decreased significantly over the period from 1980 to 2002, heavy alcohol use did not show the same decline. In fact, heavy alcohol use increased significantly from 1998 to 2002 for the first time since 1988 (Bray et al. 2003). In 2002, 27 percent of young adults (i.e., 18- to 25-year-olds) in the military reported heavy drinking, compared with only 8.9 percent of 26- to 55-year-olds (Bray et al. 2003).

Heavy drinking also is prevalent among those entering the military. A study tracking high school students into adulthood found that those who entered the military were more likely than other young adults to have been heavy drinkers in high school (Bachman et al. 1999).

When controlling for marital status, living arrangements, pregnancy, and parenthood, military service itself seemed to contribute to the increases in drinking. A study of young adults entering the U.S. Navy in 1998 examined the degree to which their drinking patterns changed from pre-entry through the first 3 years of service (Ames et al. 2004). Before entering the military, approximately 26 percent of recruits (average age 19) reported frequent heavy drinking (i.e., consuming at least five drinks [for men] and at least four drinks [for women] per typical drinking occasion at least once a week throughout the previous year) (Ames et al. 2002*a*). At followup 2 years later, the overall prevalence of frequent heavy drinking (23 percent) within the study’s cohort remained largely unchanged (Ames et al. 2004). The study found that 2 years into their military enlistment, heavy drinkers could be classified in near-equal numbers as (1) those who were pre-enlistment heavy drinkers and continued the same drinking pattern, and (2) those who were not pre-enlistment heavy drinkers but began heavy drinking after completing their training (Ames et al. 2004). These findings suggest that young adults in the military are at risk for alcohol-related problems, making them important candidates for alcohol-related prevention programs.

This article reviews the prevalence of alcohol use among young adults in all four branches of the military, comparing their drinking rates with those of young adult civilians, whether or not they are enrolled full-time in college. Risk factors for heavy drinking among young adults in the military are discussed, along with strategies for reducing hazardous drinking among these young people.

## Rates of Alcohol Use Among Young Military Personnel

Rates of heavy alcohol use among 18- to 25-year-old military personnel differ significantly by service branch and by gender, as shown in the accompanying table. For example, young males in the Marines Corps have the highest rate of heavy alcohol use, at 38.6 percent; among males in the Air Force, the rate is 24.5 percent. Young men in the Army and Navy have similar rates of heavy drinking (Army: 32.8 percent, Navy: 31.8 percent). A somewhat different pattern of heavy drinking rates is observed for young women. Rates of heavy drinking are higher for women in the Marine Corps (12.9 percent) and Navy (11.5 percent) and lower in the Air Force and Army (6.3 percent in each). Rates of heavy drinking in all service branches are nearly four times higher among young men (32.2 percent) than among young women (8.1 percent). In addition, more than half (53.8 percent) of all young military personnel reported at least one episode of binge drinking (defined here as having consumed five or more drinks on the same occasion at least once in the past 30 days) (Bray et al. 2003).

In terms of alcohol-related problems, Bray and colleagues (2003) found that the highest levels of negative effects—serious consequences (e.g., missing a week or more of duty because of a drinking-related illness or being arrested for driving while impaired^1^), productivity loss, and dependence symptoms—occurred among military personnel in the lowest pay grades (i.e., E1 to E3). These pay grades generally correspond to the youngest enlisted service members, who typically lack a college education. During 2002, 20.2 percent of junior enlisted personnel reported serious alcohol-related consequences, 27.2 percent reported lost productivity, and 22.6 percent reported symptoms of dependence.

### Military vs. Civilian Alcohol Use

The prevalence of heavy alcohol use among young military personnel differs markedly from that of civilians in the same age group, as revealed by standardized comparisons. Standardization is a set of techniques used to remove, as far as possible, the effects of differences in age, gender, or other confounding factors when comparing two populations (Last 1988). As the table shows, young men in each service branch had significantly higher rates of heavy drinking than their civilian counterparts. Of the young men in all branches of the military, 32.2 percent engaged in heavy drinking, compared with 17.8 percent of civilian men. Women serving in the Navy and the Marine Corps had significantly higher rates (11.5 percent and 12.9 percent, respectively) than civilian women (5.5 percent); rates among women in the Army and Air Force (6.3 percent in each) did not differ significantly from those of their civilian counterparts.

The figure accompanying this article highlights military–civilian differences in two types of hazardous drinking among young adults: heavy drinking and binge drinking. This figure compares rates of hazardous drinking for young adults (18–25 years old) in the military, civilian young adults (18–22 years old) enrolled full-time in college, and civilian young adults not enrolled full-time in college. The military data are from Bray and colleagues (2003), and the civilian data were obtained from the 2001 National Household Survey on Drug Abuse (Substance Abuse and Mental Health Services Administration [SAMHSA] 2002). Rates of both heavy drinking and binge drinking are higher among young adults in the military than young adult civilians, regardless of their college enrollment status. It should be noted that heavy drinking was defined slightly differently in the military and civilian surveys, and the civilian age group (18–22) was smaller than the military age group (18–25). These differences may explain some of the variability found in the rates that were compared.

**Table:** Standardized Comparisons of the Prevalence of Heavy Alcohol Use^a^ Among 18- to 25-Year-Old Military Personnel and Civilians, Past 30 Days, by Gender, 2001–2002

Gender	Comparison Population					

	Civilian	Total DOD	Army	Navy	Marine Corps	Air Force
Males	17.8% (0.5)	32.2% (2.3)^b^	32.8% (2.5)^b^	31.8% (3.5)^b^	38.6% (4.0)^b^	24.5% (3.2)^b^
Females	5.5% (0.3)	8.1% (1.0)^b^	6.3% (1.7)	11.5% (2.7)^b^	12.9% (2.3)^b^	6.3% (1.4)
**In Total Population**	15.3% (0.4)	27.3% (2.1)^b^	27.6% (2.4)^b^	26.0% (4.0)^b^	35.4% (4.8)^b^	19.8% (2.0)^b^

NOTE: Table entries are percentages, with standard errors in parentheses. Civilian data have been standardized to the U.S.-based military data by gender, age, education, race/ethnicity, and marital status. Data for the total Department of Defense and the individual services are U.S.-based population estimates (including personnel in Alaska and Hawaii). Estimates have not been adjusted for sociodemographic differences among services.

a Defined as consumption of five or more drinks on the same occasion at least once a week in the past 30 days.

b Significantly different from the civilian estimate at the 95-percent confidence interval.

SOURCE: Prevalence estimates for civilians were taken from the 2001 National Household Survey on Drug Abuse (Substance Abuse and Mental Health Services Administration 2002). Data for military personnel were obtained from the 2002 Department of Defense Survey of Health Related Behaviors Among Military Personnel (Bray et al. 2003).

## Risk Factors for Heavy Drinking Among Young Military Adults

The likelihood of heavy drinking was significantly higher after adjusting for six sociodemographic variables (Bray et al. 2003): branch of service (Army and Marine Corps personnel compared with Air Force personnel); gender; race/ethnicity (non-Hispanic Whites compared with non-Hispanic African Americans and those in the “other” racial/ethnic category^2^); education (those with a high school education or less compared with college graduates); age (those ages 21 to 25 compared with those age 35 or older); and marital status (those who were single or married with their spouse absent compared with those who were married with their spouse present). In addition to these sociodemographic risk factors for heavy drinking among military personnel, the military’s workplace culture and alcohol availability also may influence drinking practices in this population.

### Workplace Culture

Research has shown that groups of people who work together, whether in small teams or larger organizations, develop shared beliefs and practices that can influence alcohol use (see Trice and Sonnenstuhl 1990; Ames and Janes 1992). Workplace culture in the military, just as in other occupations, can be a risk factor for heavy alcohol use. For example, the workplace culture can influence beliefs about acceptable drinking contexts (most notably, drinking rituals with coworkers before, during, or after work); as well as drinking behavior (e.g., number of drinks, openly showing the effects of alcohol, getting into fights, arguing with supervisors, sleeping on the job, coming to work with a hangover) (Ames et al. 1997); and expectations about the positive or negative consequences of drinking (Grube et al. 1994).

Ames and colleagues (2004) recently studied the influence of workplace culture on drinking practices in various military settings of the Navy. Face-to-face interviews with young Navy personnel revealed established drinking rituals and routines as well as elements of the work environment that encouraged drinking at work on land bases and during deployment liberties (i.e., shore leave). For example, young sailors viewed drinking with coworkers during the work week as an appropriate coping mechanism in response to stress, boredom, loneliness, and lack of other recreational activities. The respondents described heavy (i.e., five drinks or more for men and four drinks or more for women per typical drinking occasion) and binge drinking behavior after work, and especially drinking on liberty during deployment, as part of a cultural tradition. (Binge drinking was defined in this study as five drinks or more for men and four drinks or more for women *within a 2-hour period*.) On deployment liberty, binge drinking and drinking to the point of intoxication were not necessarily viewed as inappropriate or punishable behavior, unless sailors were too intoxicated to return to ship at the designated time. The researchers found that cultural norms for drinking during shore leave^3^ were significantly associated with frequent heavy drinking, the number of days on which binge drinking occurred, and the average amount of alcohol consumed daily (Ames et al. 2004).

### Alcohol Availability

Another factor that may influence heavy and binge drinking among young adults in the military is the physical and social availability of alcohol. Alcohol availability is a known risk factor for increased alcohol use in the general population (Gruenewald et al. 1993) and in occupational settings (Ames and Grube 1999). For example, the personnel interviewed in the Navy study reported that alcohol and opportunities for drinking were easily available both in foreign ports (where the U.S. minimum legal drinking age usually does not apply) and on U.S. bases. On base, beer and spirits are stacked for display at the entry to the post exchange. Navy underage recruits reported that they had easy access to alcohol in bars, in the barracks, or in hotel rooms near the base. On shore leave in foreign ports, alcohol was reportedly inexpensive, bars were located near the point of disembarkation, few ports had underage drinking laws, and most sailors who wanted to drink organized drinking groups before disembarking (Ames et al. 2004).

**Figure:**
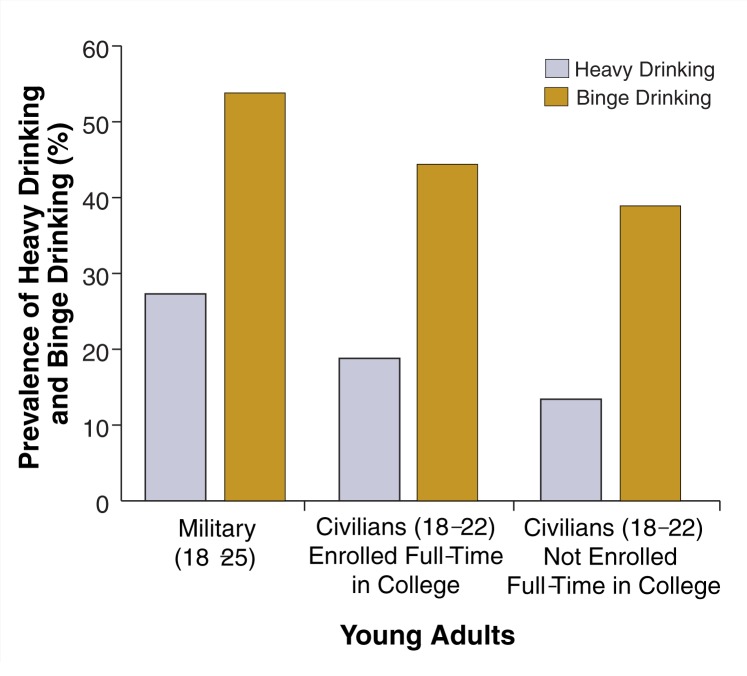
Hazardous drinking among young adults in the military and civilians, by college enrollment status, 2002. SOURCES: Military data: Bray et al. 2003. Civilian data: Substance Abuse and Mental Health Services Administration 2003.

## Strategies to Prevent Alcohol Problems

As demonstrated in the Navy study, factors contributing to alcohol use among young adults in the military may include established drinking cultures that feature drinking rituals and traditional celebrations, expectations about heavy drinking after work and while on leave, drinking to cope and as a recreational activity, and the social and physical availability of alcohol. Strategies to prevent alcohol misuse and related problems in this population, therefore, may attempt to target these factors.

Current strategies to prevent alcohol problems among military personnel include instituting and enforcing policies that regulate alcohol availability and pricing, deglamorizing alcohol use, and promoting personal responsibility and good health.

Although the U.S. military has implemented policies and programs designed to reduce alcohol use and related problems among personnel, examples of which are described below, there has been little formal evaluation of these programs. This is an important area for future research, as evaluation is a critical component of any comprehensive prevention program. Thus, despite the military’s efforts to address problem drinking, the effectiveness of these programs in reducing or preventing hazardous drinking and its attendant problems remains largely unknown. The programs described below are representative of the military’s prevention efforts; future research will be needed to evaluate their utility.

### Alcohol Use Policies

For many work organizations, an important strategy for minimizing alcohol problems among employees is the establishment and enforcement of workplace alcohol use policies. Such policies can help to change the characteristics of the workplace culture or other social environments that support heavy and binge drinking. The U.S. military adopted strict policies in the early 1980s with the aim of reducing rates of alcohol, tobacco, and illicit drug use (Department of Defense [DOD] 1980). These policies included components for detection, treatment and rehabilitation, and prevention. An analysis of survey data from 1980 to 1995 found lower rates of illicit drug use among military personnel relative to civilians, suggesting that the policies were effective in this regard (Bray and Marsden 2000). However, such differences were not found in the rates of alcohol use, especially when accounting for demographic changes in the military, suggesting that the substance use policies have not been effective in reducing alcohol use (Bray and Marsden 2000).

In another study, Bachman and colleagues (1999) compared changes in substance use rates among young military recruits before and 2 years after enlistment with changes in use rates among their civilian counterparts. Examining trends in these comparisons across two decades, the researchers found that rates of illicit drug use decreased more among the military recruits than among civilians, especially after the military implemented mandatory routine drug testing in 1980. Although the rates of heavy drinking also decreased over the past two decades for both military recruits and civilians, the researchers did not find sufficient differences in the rates to indicate that military alcohol policies have been particularly effective (Bachman et al. 1999).

In contrast, Voas and colleagues (2002) reported findings on the positive effects of a specific policy change designed to reduce off-base alcohol use among young marines stationed near the Mexican border. Marines stationed at Camp Pendleton, California, 67 miles from the Mexican border, were drawn to the bars in Mexico by inexpensive alcohol and a minimum drinking age of 18, and often returned to base on weekend nights with high blood alcohol contents (BACs). In response, commanders at Camp Pendleton adopted a policy that required marines to receive written permission to cross the border. After the policy was implemented, the number of underage marines returning across the Mexican border was reduced by 78 percent, and the number returning with BACs of 0.08 percent or higher was reduced by 84 percent. The authors note that several elements of the policy change may have contributed to the outcome. For example, marines applying for permission to cross the border received information warning of the possible problems involved, including the potential for disciplinary action. The fact that the new policy required more effort and planning by the young marines also may have served as a deterrent (Voas et al. 2002).

### Alcohol Pricing

Research suggests that alcohol use and related problems are reduced when alcoholic beverage prices are increased (Cook and Moore 2002). DOD policies, however, allow alcoholic beverages sold in military stores to be discounted below prices in local civilian stores. The DOD’s Alcohol Abuse Prevention Strategic Plan states that alcoholic beverages in military stores should be priced at no more than 5 percent below the local competitive price, except in States with alcohol beverage control boards, where prices should be no more than 10 percent below the local competitive price (DOD 1999). A 1997 review by the Office of the Inspector General found that these policies were used to set store prices and that patrons of military retail stores benefited from additional discounts because of the stores’ exemption from sales tax. The review reported, for example, that the prices on beverages sold at a military retail store in one area ranged from 9 percent to 27 percent less than prices in State-operated alcohol stores. The authors concluded that the DOD’s pricing policy was inconsistent with its policy for maintaining a healthy active-duty force, and they recommended that prices in military stores equal those charged in the commercial retail market (Office of the Inspector General 1997).

### Alcohol Use Deglamorization

All branches of the U.S. military have made efforts to deglamorize the use of alcohol, providing nonalcoholic beverages at functions where alcohol is served and emphasizing that alcohol use before or during work hours is unacceptable. The Navy’s “Right Spirit” campaign calls for removing alcohol from traditional ceremonies, providing alternatives to drinking, recognizing the effects of alcohol use, and promoting personal responsibility concerning alcohol use (Bickford et al. 2004). The Navy Alcohol and Drug Abuse Prevention Program credits this campaign for a nearly 40-percent reduction in alcohol-related incidents (i.e., infractions in which alcohol played a role) from 1996 to 2000, and for a nearly 50-percent decline in arrests for driving under the influence (U.S. Navy 2005).

A survey of Air Force officers attending Air Command and Staff College (ACSC) sought to determine the degree to which the Air Force deglamorization campaign is reflected in the alcohol use norms of ACSC students. Survey respondents generally agreed that the ACSC environment is supportive of alcohol deglamorization but noted that despite the deglamorization efforts, students’ attention often is focused on alcohol, bringing alcohol to social activities is emphasized, and the student population does not consistently view drinking during the workday as unacceptable (Lyman 1999). The author recommended that the ACSC command structure and faculty support the deglamorization campaign by continuing to emphasize responsible alcohol use, encouraging the use of designated drivers, and recommending alcoholism treatment when necessary.

### Personal Responsibility Campaigns

Alcohol policies in the military often emphasize each individual’s personal responsibility regarding alcohol use. For example, alcohol policies in the Navy emphasize “responsible use” or “the application of self-imposed limitations of time, place, and quantity when consuming alcoholic beverages” (Bickford et al. 2004). The Navy’s “Best Practices” program aims to reduce alcohol and other drug problems among young at-risk personnel by stressing relationships, relevance, and responsibility. The program emphasizes the impact of each person’s behavior on the organization. It encourages those in command to foster positive professional relationships with sailors and focuses on the responsibility of leaders to ensure that sailors live and work in an environment conducive to learning. The program also focuses on the responsibility of sailors to learn and understand Navy policies and expectations. Other elements of the program include providing alternative activities to engage sailors during free time, education sessions including drug abuse awareness training, and consistent use of discipline in response to violations (see the Web site of the Navy Alcohol and Drug Abuse Prevention Program at http://navdweb.spawar.navy.mil/index.htm).

Another Navy program, PREVENT (Personal Responsibility: Values and Education Training) aims to provide 18- to 26-year-old sailors with the education and skills necessary to encourage them to act as personally responsible, contributing members of the Navy. Evaluations are not formalized, but the findings reported by the Navy indicate that sailors who attended PREVENT sessions had fewer binge drinking episodes compared with their pre-enlistment frequencies and showed a greater personal awareness and responsibility for their alcohol use patterns and consequences (U.S. Navy 2004).

### Health Promotion Programs

In 1986, the U.S. military adopted a comprehensive policy to foster general health promotion among military personnel, including strategies to reduce substance abuse within this directive. In addition to alcohol and other drug abuse prevention, the policy included measures for smoking prevention and cessation, physical fitness, nutrition, stress management, and the prevention of hypertension (DOD 1986). A 1991 survey of Army personnel found that those who reported hazardous drinking (defined as 21 or more drinks per week for men, 14 or more for women) were more likely than other drinkers to engage in risky behaviors such as speeding, tobacco use, and not wearing seat belts. Hazardous drinkers tended to be younger and were less likely to be married. The authors suggest that, given the association between hazardous drinking and other risky behaviors, incorporating alcohol abuse prevention into general health promotion programs may be an especially effective strategy for this population (Fertig and Allen 1996).

## Summary

As surveys of military personnel indicate, heavy alcohol use remains a problem in this population. Those especially likely to report heavy drinking are young non-Hispanic White men, with a high school education or less, who are either single or married but living away from their spouse. Military personnel report that drinking often is used to cope with stress, boredom, loneliness, and the lack of other recreational activities. The easy availability of alcohol and drinking opportunities also contributes to alcohol use in this population.

Military alcohol use policies combined with campaigns focusing on alcohol deglamorization, personal responsibility, and health promotion may help reduce heavy alcohol use in this population, but further research is needed to evaluate the effects of these measures. The recent increase in heavy drinking seen throughout the military suggests that these efforts have not been successful in countering hazardous drinking behavior. Most importantly, more research is needed to gain greater understanding of the characteristics of the military culture that promote or allow heavy drinking and binge drinking practices. Based on findings from solid empirical research, the next step would be to develop systemwide approaches to modifying the aspects of the military environment that show the most promise for lowering alcohol consumption rates among young adults, thereby reducing the prevalence of heavy and binge drinking and related problems to a level far below those reported in this paper.

## Footnotes

1 Other serious consequences included not being promoted, receiving a low performance rating, being arrested for another alcohol-related reason, being involved in a traffic crash resulting in injury or property damage, and fighting while drinking.

2 The “other” category includes all people not classified elsewhere, such as Native Americans or Asians.

3 Cultural norms were based on the respondent’s perceptions about whether his or her supervisor, friends, or coworkers would disapprove or approve of drinking during shore leave and how many drinks the respondent thought his or her supervisor, friends, or coworkers usually had when they drank.
